# Maxillary Reconstruction Using a Gullwing Fibula Osteofascial Flap and Flexor Hallucis Longus Muscle

**DOI:** 10.1097/GOX.0000000000001821

**Published:** 2018-06-11

**Authors:** Stuart L. Mitchell, Akhil K. Seth, Evan Matros, Peter G. Cordeiro

**Affiliations:** From the * Department of Orthopaedic Surgery, The Johns Hopkins University School of Medicine, Baltimore, Md.; † Plastic and Reconstructive Surgical Service, Department of Surgery, Memorial Sloan Kettering Cancer Center, New York, N.Y.

## Abstract

The appropriate reconstruction of an infrastructure maxillectomy defect requires vascularized bone for maxillary arch restoration, soft tissue bulk for filling the residual defect extending up to the orbital floor, and a thin tissue layer for resurfacing the palate and adjacent cheek mucosa. Although several free tissue flaps have been previously described as reconstructive options, each possesses limitations. We describe the fibula osteofascial flap with flexor hallucis longus muscle, no skin paddle, and a “gullwing” fascial component, as an ideal reconstructive option for these specific maxillary defects. It satisfies the necessary requirements of bone, restoration of intraoral surfaces, as well as additional soft tissue volume to provide the optimal aesthetic and functional result. It also has the added benefit of minimizing morbidity to, and improving aesthetics of, the donor site. This is demonstrated through a case presentation and review of the existing literature.

## INTRODUCTION

Reconstruction of the midface following tumor extirpation presents unique challenges to the reconstructive surgeon. For infrastructure maxillectomy defects (Cordeiro Type 2^1^), maximizing aesthetic and functional outcomes requires: (1) well-vascularized bone for maxillary arch restoration; (2) adequate soft tissue bulk to fill above the arch and palate; (3) thin, durable tissue to resurface the palate and anterolateral gingivobuccal sulcus. The radial forearm osteocutaneous, scapular osseous, and fibula flaps have been the principal flaps described for reconstructing these specific defects.^2–5^ In particular, the bone-only^6^, osteoadipofascial^7^, and osteocutaneous fibula flaps^2,3^ have all been utilized, but often provide either insufficient or excessive soft tissue for surface lining. Meanwhile, the osteomyofascial fibula flap^8^ can provide fascia that is appropriate for lining, but can lack the volume necessary for obliterating the space above the reconstructed bone.

We describe the fibula osteofascial flap, with flexor hallucis longus (FHL) muscle and a “gullwing” fascial component, as an ideal alternative for Type 2 defect reconstruction. This newly described flap provides an ideal combination of bone, soft tissue bulk, and mucosal resurfacing that optimizes reconstruction, while minimizing donor-site morbidity by avoiding the harvesting of a skin paddle.

## CASE

The patient was an otherwise healthy 54-year-old male with stage 1, T1N0, squamous cell carcinoma who previously underwent partial right upper alveolectomy and obturator reconstruction 3 years prior. Two years postoperatively, he developed a local recurrence, requiring definitive upper alveolectomy including bone and hard palate resection from the midline to the right posterior molar. There was no history of radiation or plan for adjuvant therapy. Reconstructive options were reviewed including an osteocutaneous radial forearm or fibula flap, depending on the final intraoperative defect size.

## TECHNICAL DESCRIPTION

**Fig. 1.**
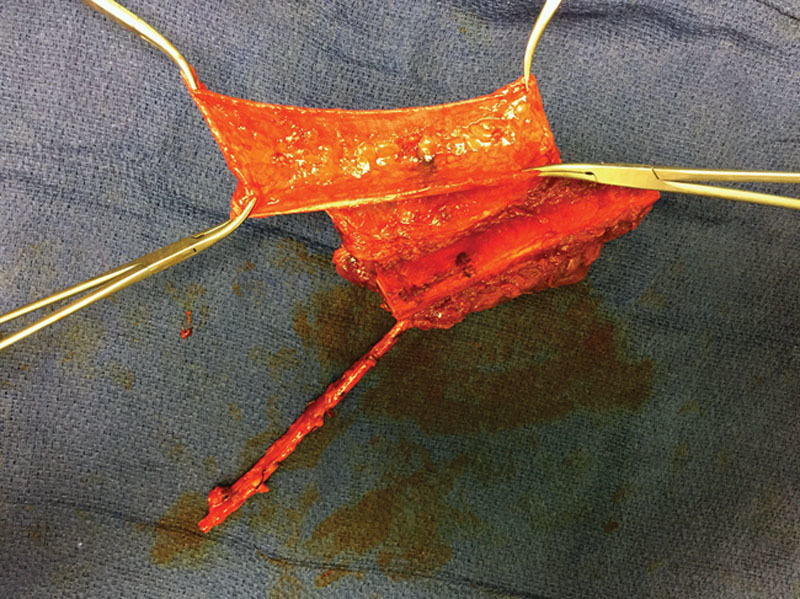
Intraoperative image demonstrating fibula flap raised with preservation of fascia extending from septum of flap. Flap is tailored to size with demonstration of the gullwing configuration of the fascia off of the septum, the flexor hallicus longus muscle, and the vascular pedicle.

**Fig. 2.**
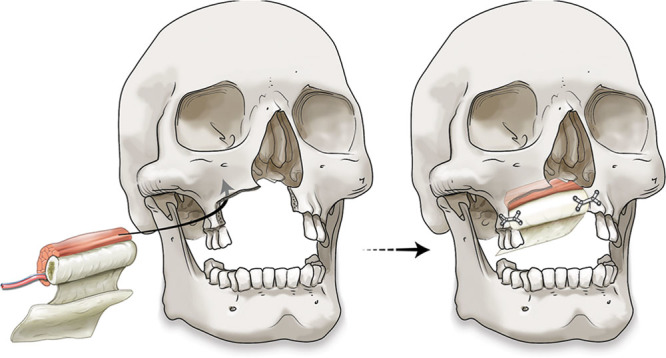
Schematic representation of orientation of flap before and after inset with titanium mini-plates.

The residual defect extended from the right posterior molar across midline to the left lateral incisor, leaving a relatively small palatal defect, but a large bony gap that required a fibula flap. To avoid a bulky intraoral skin island, a fascia-only flap was raised based on the interosseous septum (Figs. 1, 2). A straight-line skin incision was made along the posterolateral aspect of the fibula and suprafascial skin flaps were elevated anteriorly and posteriorly off of the lateral and posterior compartment musculature. The fascia was incised circumferentially and raised off the muscle, yielding an 8 × 15-cm fascial flap that was centered on the intermuscular septum in a gullwing configuration. The osseous portion of the flap was elevated in the standard fashion, including removal of the lateral and anterior compartment musculature off the bone, concurrent harvesting of the FHL, and osteotomies 15-cm above the ankle joint and 4-cm below the fibular head.

**Fig. 3.**
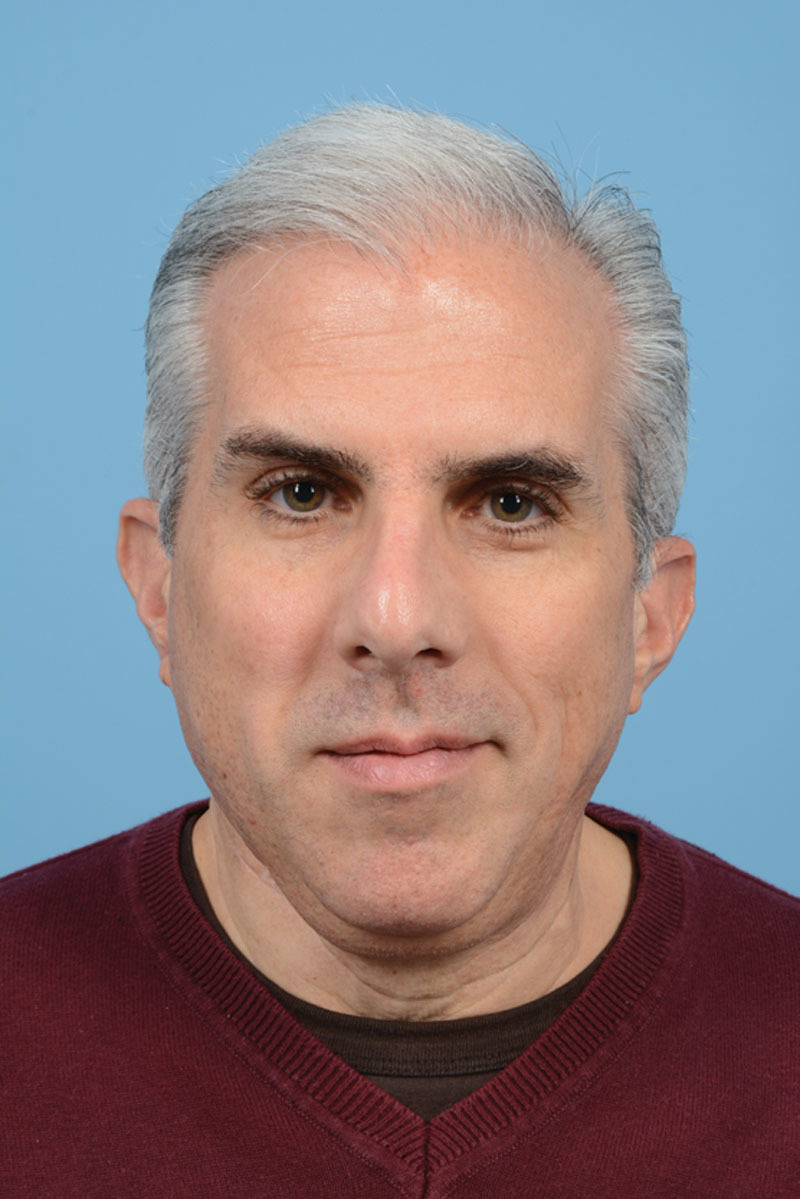
Clinical photograph of patient 6 months postoperatively demonstrating good facial symmetry.

**Fig. 4.**
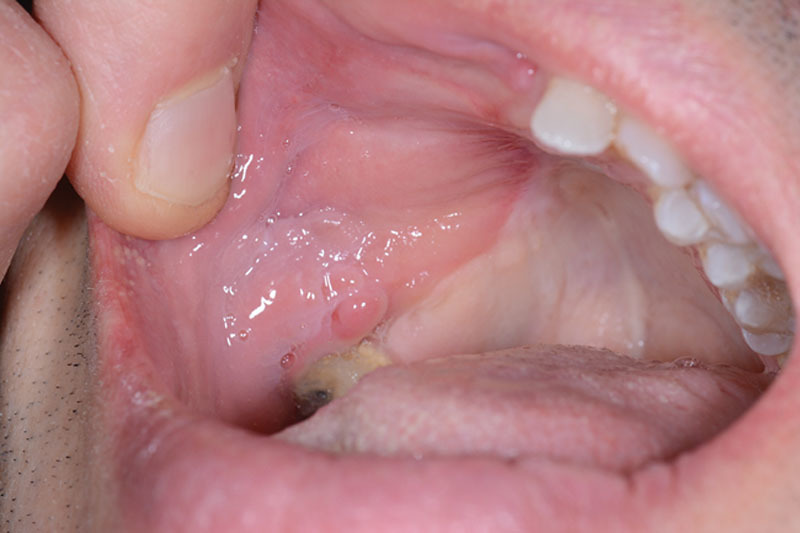
Clinical photograph 6 months postoperatively demonstrating good mucosalization of the fascial component of the flap intraorally.

The flap was harvested and transferred to the neck for inset and microvascular anastomosis. Two additional fibula osteotomies were made and then inset into the maxillary defect using titanium mini-plates and screws. The flap pedicle was tunneled subcutaneously to the neck where the external carotid artery and external jugular vein were used for microvascular anastomosis. The FHL was utilized to fill the defect above the bone extending up to the orbital floor. The fascial flap was then laid over the FHL to reinforce the palatal closure, with 1 wing inset to the mucosal edge just past midline and the other wing sutured laterally to the cheek mucosa. Postoperatively, the patient did well with advancement of his diet on postoperative day 10 and no breakdown of his palatal closure (Figs. 3, 4). He is currently undergoing preparation for placement of osseointegrated implants.

## DISCUSSION

We have previously described a classification system and reconstructive algorithm for defects of the maxilla and midface.^1,9,10^ Type 2 maxillary defects are subtotal maxillectomies that involve the palate and maxillary bony arch but leave the orbital floor and upper maxilla intact. Although a bony strut is required to restore maxillary arch structure, this defect has relatively small volume and surface area requirements. A “sandwiched” osteocutaneous radial forearm flap can meet these reconstructive goals^5^; however, it is not an ideal donor site and its bone stock can often be insufficient for dental implant placement. Others have described using an osteocutaneous fibula flap for these defects,^3^ but the cutaneous portion of the flap is often too thick and projects into the oral cavity.^7,8^ In contrast, the bone-only^6^ and osteoadipofascial^7^ fibula flaps may lack the soft-tissue volume needed for re-lining the palate and cheek mucosa. The fibula can also be harvested in combination with muscle, including the FHL, peroneus longus, or soleus, and then inset with muscle sutured to the palatal defect edges. Although the exposed muscle can mucosalize, in our experience it often contracts and atrophies, leading to problematic palatal fistulae. Fan et al.^8^ describe an osteomyofascial fibula flap,^8^ which maintains a small cuff of soleus and peroneal muscles on the fibula periosteum that can mucosalize but may also not provide enough soft tissue bulk for filling the residual defect above the maxillary arch.

We describe a novel gullwing, osteofascial fibula flap, with FHL muscle and no skin paddle, as an ideal flap for Type 2 maxillary defect reconstruction. The fibula provides excellent quality bone for dental implant placement, while the FHL muscle belly can be contoured to the associated soft-tissue defect. When designed as a gullwing, the fascia can be harvested as large as needed to cover the muscle, providing the watertight closure necessary to prevent wound breakdown or hardware exposure. Consequently, large intraoral areas can be resurfaced without a skin graft for the fibula donor site. This allows for a significantly improved donor-site aesthetic, faster patient mobilization, and straightforward donor-site care postoperatively.

Although this patient was not radiated, this flap can be used with neoadjuvant and adjuvant radiotherapy. In this setting, a flap with muscle and fascia is preferable to bone alone. Meanwhile, having a flap skin island, although providing a superior mucosal closure, can often be too bulky intraorally. In contrast, the osteofascial flap fascia provides excellent tissue for reconstructing the adjacent palatal and cheek mucosal defects, restoring the buccogingival sulcus while contributing minimal tissue volume as compared with a cutaneous paddle.
